# Surveying Holistic Well-Being for Work from Home Employees: Insights for Organizational Practices

**DOI:** 10.1080/24725838.2025.2524778

**Published:** 2025-07-06

**Authors:** Yifan Li, Jess Francis-Levin, Isabelle German

**Affiliations:** a Department of Industrial and Operations Engineering, University of Michigan, Ann Arbor, MI, USA; b Institute for Social Research, University of Michigan, Ann Arbor, MI, USA

**Keywords:** Total worker health, WFH, remote work, NIOSH WellBQ

## Abstract

**Background:**

Due to the COVID-19 Pandemic, many traditional work settings have shifted to Work from Home (WFH). In traditional work modalities, interactions are more controlled, and tools can be standardized. In a WFH environment, the interactions and tools accessible become less well-defined. In addition, home-life and work-life start to blend. Existing literature suggests mixed impacts on WFH workers. Positive impacts include lower stress, better productivity, and more flexibility to shift between work-life and home-life. However, negative impacts include worse physical health, worse work-life balance, and increased social isolation. Thus, it is important to understand the overall well-being of WFH workers and identify the well-being challenges that different demographic groups face.

**Purpose:**

The aim of this study was to evaluate the well-being of a WFH population and identify differences between demographic groups. The NIOSH Worker Well-Being Questionnaire (WellBQ) was used to evaluate the well-being of individual workers.

**Methods:**

The WellBQ was input into Qualtrics and distributed through a University of Michigan recruitment pool. After six weeks, a total of 329 responses were collected and scored following the methods outlined in the WellBQ. Point-biserial correlations were performed to identify trends in well-being versus demographic variables.

**Results:**

We found statistically significant correlations between various factors in all five major WellBQ categories and demographic variables. Those with higher income and higher education, had higher productivity and mental health related well-being scores, but typically scored lower in overall health.

**Conclusions:**

Through review of survey data, we scored the holistic well-being of WFH employees along different factors, and identified demographic variables associated with differing levels of health. This suggests that each WFH demographic group experienced their own pros and cons, and employers need tailored resources and tools to support all workers.

## 1. Introduction

The COVID-19 pandemic changed many aspects of life, including work and the increase of Work from Home (WFH). Over half the U.S. workforce now works remotely at least once weekly (Parker et al., 2022). This shift prompted new work modalities, changing how individuals engage with work environments and colleagues, and a blending of work, social, and home lives. It also highlighted the need to understand barriers to WFH, including access to digital tools, skills required to use them effectively, and well-being support. The digital divide refers to how individuals with higher education and income typically have better access to and skills with digital tools (Van Dijk & Hacker, 2003). WFH deepens this divide, as remote work is more accessible to those with higher education and income (Bergefurt et al., 2023; Budnitz & Tranos, 2022; Soga et al., 2024).

Prior to the pandemic, WFH modalities did exist, involving a smaller percentage of workers (~10% in the USA). Most studies involving these cohorts indicated positive outcomes (Hackney et al., 2022). With the increase in WFH caused by Covid-19, research suggests that remote or hybrid settings can improve work-life balance, well-being, and productivity compared to traditional work (Hackney et al., 2022; Parker et al., 2020, 2022; Wells et al., 2023). However, drawbacks of WFH include struggles with weight gain, physical discomfort, technology fatigue, and social isolation (Bailenson, 2021; Streeter et al., 2021; Wells et al., 2023). Blurred boundaries between home and work have amplified distractions, increased caregiving demands, disrupted work-life balance, and heightened stress (Galanti et al., 2021; Parker et al., 2022; Prasad et al., 2020). Workers without dedicated home offices report more distractions, higher stress, poorer ergonomics setup for their home workstation, and lower overall well-being (Bergefurt et al., 2023; Hackney et al., 2022; Wells et al., 2023). Increased connectivity to work can also lead to fewer breaks and more physical discomfort (Cropley et al., 2022). Interestingly, more negative impacts of WFH were noticed during the pandemic compared to prior (Hackney et al., 2022).

With the prevalence and persistence of WFH, understanding the holistic well-being of remote workers is essential as both positive and negative impacts have been reported in literature. Our goal in this study was to identify well-being differences among WFH workers from different demographic backgrounds. Identifying well-being differences could provide insights on organizational support systems for the disadvantaged demographic groups.

## 2. Methods

This study focused on evaluating WFH worker well-being using the NIOSH Worker Well-Being Questionnaire (WellBQ), a tool designed to measure well-being as a holistic construct (Chari et al., 2022; NIOSH, 2021). We administered the WellBQ to workers in fully remote or hybrid (minimum 1 day weekly remote) environments and evaluated demographic divides to examine potential well-being barriers within WFH workers. One attention-check question was added to ensure engagement. Those under 18 or not performing WFH were screened out. The study received Institutional Review Board approval at the University of Michigan (HUM00222242). The instrument was input into Qualtrics as an online survey, using WellBQ recommended scoring. We employed purposive sampling, utilizing an online recruitment pool (*N* > 83,000) managed and hosted by the University of Michigan, and kept it open for 6 wk in 2023. All participants were provided informed consent and indicated willingness to participate.

### 2.1. Survey Scoring

The WellBQ has 68 questions (estimated thirty minutes) consisting of multiple-choice and matrix questions. Each question contributes to one of 52 factors under five categorical scores: 1) Work Evaluation and Experience; 2) Workplace Policy and Culture; 3) Workplace Physical Environment and Safety Climate; 4) Health Status; and 5) Home, Community, and Society. Scoring was directly adopted from the WellBQ. In matrix questions, responses were averaged across the line items (NIOSH, 2021). In addition, certain item reflected high scores as positive. For example, Q2 in the WellBQ measures wage satisfaction: ‘Not at all satisfied’ scores 1 (negative), while ‘Very satisfied’ scores 4 (positive). Other items reflected low scores as positive. For example, Q47 asks about the number of poor mental health days: ‘Not at all’ scores 1 (positive), while ‘Nearly every day’ scores 4 (negative). Thus, some factors have a high score indicating positive outcomes, while others have lower scores indicating positive outcomes.

### 2.2. Statistical Analysis

The factors in the five categories were tested against demographics: age, gender, income, education, and WFH level. A point-biserial correlation was run with an alpha level of 0.05. Demographics were binned into high versus low to simplify analysis; cutoff levels were chosen to minimize skew between high and low. Age was split at 45 years, income at $75k annually, education based on college completion, and WFH level binned as high if 4 or more days.

## 3. Results

We received 329 responses with 246 analyzed after screening for attention check, age, and WFH modality. The numbers of participants across demographic factors are shown in Appendix A, Table A1. During analysis, further responses were dropped if the participant missed needed information. Summary statistics for all variables across the entire population, as well as split by demographic variables, and correlations, are presented in Appendix B, Tables B1–B5.

Within ‘Work Evaluation & Experience’, the following were significant. Higher income correlated with higher coworker support (*r*(241) = 0.22, *p* < 0.001), benefits satisfaction (*r*(241) = 0.17, *p* = 0.008), negative emotions (*r*(240) = 0.14, *p* = 0.027), job satisfaction (*r*(241) = 0.23, *p* < 0.001), wage satisfaction (*r*(240) = 0.28, *p* < 0.001), autonomy (*r*(241) = 0.19, *p* = 0.003), work meaning (*r*(241) = 0.14, *p* = 0.035), and positive emotions (*r*(239) = 0.18, *p* = 0.006). Higher education correlated with higher benefits satisfaction (*r*(243) = 0.18, *p* = 0.01). Higher age correlated with higher negative emotions (*r*(243) = 0.14, *p* = 0.029), wage satisfaction (*r*(243) = 0.17, *p* = 0.008), autonomy (*r*(244) = 0.22, *p* < 0.001), and meaning at work (*r*(244) = 0.20, *p* = 0.002). Females experienced higher autonomy (*r*(242) = 0.13, *p* = 0.044) and meaning at work (*r*(242) = 0.13, *p* = 0.036). Higher level of WFH correlated with a higher perception of autonomy (*r*(244) = 0.15, *p* = 0.019).

Within ‘Policies & Culture Category’, the following were significant. Higher income correlated with better rated health culture (*r*(241) = 0.04, *p* = 0.02) and job benefits (*r*(236) = 0.11, *p* < 0.001). Higher education correlated with better job benefits (*r*(238) = 0.15, *p* = 0.01), better health culture (*r*(243) = 0.14, *p* = 0.02), and being less likely to bring non-work conflicts to work (*r*(243) = −0.14, *p* = 0.02). Higher WFH levels correlated to being less likely to bring non-work conflicts to work (*r*(244) = −0.17, *p* < 0.001).

Within ‘Physical Environment & Safety Climate’, the following were significant. Higher income correlated to higher occurrences of discrimination (*r*(240) = 0.16, *p* = 0.010), witnessing physical violence (*r*(240) = 0.16, *p* = 0.011), and sexual harassment (*r*(239) = 0.14, *p* = 0.024). Higher education correlated with higher occurrences of witnessing physical violence (*r*(242) = 0.15, *p* = 0.02), sexual harassment (*r*(241) = 0.29, *p* < 0.001), discrimination (*r*(242) = 0.19, *p* < 0.001), and rates of bullying (*r*(241) = 0.16, *p* = 0.01). Higher age correlated with lower perceived safety climate (*r*(241) = −0.13, *p* = 0.038), higher discrimination (*r*(243) = 0.14, *p* = 0.025) and higher occurrences witnessing physical violence (*r*(243) = 0.13, *p* = 0.034). Females experience lower perceived safety climate (*r*(239) = −0.18, *p* = 0.004) and higher occurrences witnessing physical violence (*r*(241) = 0.15, *p* = 0.014). Higher WFH levels experience lower perceived safety climate (*r*(241) = −0.25, *p* < 0.001).

Within ‘Health Status’, the following were significant. Higher income correlated with worse nutrition (*r*(217) = −0.14, *p* = 0.037), worse health overall (*r*(241) = −0.14, *p* = 0.026), lower health limitations (*r*(241) = −0.19, *p* = 0.003), lower tobacco use (*r*(239) = −0.23, *p* < 0.001), less sleepy at work (*r*(241) = −0.14, *p* = 0.022), lower stress (*r*(236) = −0.23, *p* < 0.001), lower poor mental health days (*r*(228) = −0.22, *p* < 0.001), higher productivity (*r*(236) = −0.21, *p* < 0.001), lower cognitive limitations (*r*(241) = −0.26, *p* < 0.001), and lower occurrence of poor mental health (*r*(237) = −0.29, *p* < 0.001). Higher education levels correlated with worse overall health (*r*(243) = −0.15, *p* = 0.02), less sleep (*r*(243) = −0.16, *p* = 0.01), lower occurrence of insomnia (*r*(242) = −0.16, *p* = 0.01), lower cognitive limitations (*r*(243) = −0.17, *p* = 0.01), lower health limitations (*r*(243) = −0.16, *p* = 0.01), lower tobacco use (*r*(241) = −0.20, *p* = 0.002), less likely to be injured (*r*(241) = −0.14, *p* = 0.02), less chronic conditions (*r*(236) = −0.23, *p* < 0.001), and lower occurrence of poor mental health (*r*(239) = −0.22, *p* < 0.001). Higher age correlated with less risky drinking (*r*(243) = −0.12, *p* = 0.048), less tobacco use (*r*(242) = −0.14, *p* = 0.025), lower stress (*r*(239) = −0.21, *p* = 0.001), lower poor mental health days (*r*(231) = −0.19, *p* < 0.001), higher productivity (*r*(239) = −0.18, *p* = 0.004), lower occurrence of poor mental health (*r*(240) = −0.18, *p* < 0.001), and higher chronic conditions (*r*(237) = 0.21, *p* < 0.001). Being female correlated with less drinking (*r*(239) = −0.24, *p* < 0.001), less tobacco use (*r*(240) = −0.26, *p* < 0.001), and lower workplace injury (*r*(240) = −0.15, *p* = 0.013). Higher WFH correlated with more days of poor physical health (*r*(233) = 0.17, *p* = 0.009), and higher occurrence of insomnia (*r*(243) = 0.13, *p* = 0.036).

Within ‘Home, Community & Society’, the following were significant. Higher income correlated with higher financial insecurity (*r*(240) = 0.28, *p* < 0.001), higher life satisfaction (*r*(240) = 0.26, *p* < 0.001), more non-work support (*r*(241) = 0.22, *p* < 0.001), and less activities outside work (*r*(240) = −0.22, *p* < 0.001). Higher education correlated with higher life satisfaction (*r*(242) = 0.12, *p* = 0.05), higher non-work support (*r*(243) = 0.19, *p* < 0.001), and less activities outside work (*r*(242) = −0.14, *p* = 0.02). Higher age correlated with less activities outside work (*r*(243) = −0.24, *p* < 0.001). Females had more non-work support (*r*(242) = 0.16, *p* = 0.012).

## 4. Discussion

### 4.1. Demographics, Barriers & Support

With WFH, research substantiates a digital divide that favors white-collar jobs and those with higher education and income (Bergefurt et al., 2023; Budnitz & Tranos, 2022; Soga et al., 2024; Van Dijk & Hacker, 2003). The current study examined well-being for WFH employees.

Thematically, higher income correlated with well-being factors such as better support, greater health benefits, higher life satisfaction, and less impact from mental health problems. Similarly, higher education correlated with better health benefits, support, fewer chronic conditions, less mental health impact, and higher life satisfaction. These initial results suggest that individuals with higher income and education are more satisfied with their work experience, have better access to support and benefits, and report higher well-being related to productivity and mental health. Such findings align with existing literature showing that these groups typically have better access to WFH tools and support (Bergefurt et al., 2023; Budnitz & Tranos, 2022; Soga et al., 2024).

Interestingly, higher income also correlated with more negative emotions, worse overall health, worse nutrition, increased exposure to sexual and physical violence, discrimination, and higher financial insecurity. Similarly, higher education correlated with negative factors such as insomnia, worse overall health, and increased exposure to violence, discrimination, and bullying. This finding aligns with evidence of worse overall health in connection with the shift to remote work (Wells et al., 2023). WFH may impact those with higher income and education more, as they are more likely to be heavily engaged in work. Future work could examine correlations between high income/education and work hours. Some results may relate to greater work-related stress or longer hours among these participants, potentially sacrificing aspects of well-being for others, though research is needed to support this hypothesis.

Older age correlated with greater well-being factors, including better benefits, lower stress, and less impact from poor mental health. Better benefits may stem from longer tenures, while lower stress and better mental health could reflect a ‘survivor effect’, in which those remaining in the workforce longer are naturally healthier (Arrighi & Hertz-Picciotto, 1994). However, older respondents also reported lower perceptions of workplace safety, more negative feelings, and greater discrimination. These findings may result from age discrimination, which not unique to WFH workers (Bayl-Smith & Griffin, 2014). Older age also correlated with chronic health issues, expected with aging.

Females had higher work autonomy, found work more meaningful, and were less likely to be injured in the workplace. However, they also reported lower perceived safety climate at work. Those at a higher WFH level reported higher autonomy, but also lower perceived work safety climate and more physical health problems. Higher autonomy may allow these individuals to work remotely more to avoid perceived safety concerns. They may also have the flexibility to work remotely when facing physical ailments. Both hypotheses warrant further study. While sexual and physical violence did appear to be significant across multiple demographics, both questions were framed in a binary sense (0-not witnessed, 1-have witnessed), with minimal numerical differences between the groups.

Our findings suggest that employers should be purposeful in supporting WFH workers who are conventionally disadvantaged, such as those of lower income and lower education. These individuals overall reported higher stress, worse mental health, and lower productivity. Pre-pandemic studies generally indicate positive impacts of WFH, while mixed and negative impacts were reported during the pandemic due to employers being unprepared in terms of infrastructure and policies (Hackney et al., 2022). Providing home office tools, digital skills courses, and additional Human-Resources (HR) and employee assistance programs, are all avenues to improve well-being (Bergefurt et al., 2023). HR programs can also aid employees from the demographic groups who reported negative impacts of discrimination and bullying (e.g., higher age, higher income, and highly educated). Such resources may help employees maximize WFH benefits while minimizing drawbacks. Employers are also advised to take actionable steps toward encouraging work-life balance among employees to prevent the sacrifice of well-being for the sake of productivity.

### 4.2. WellBQ Tool Commentary

In the WellBQ, we observed scoring inconsistencies within larger categories. Some items scored positively with higher values, while others scored positively with lower values. This structure complicates category integration. Additionally, some WellBQ factors average multiple questions, giving each question a different weight in final scoring. The WellBQ included ‘Don’t know’ or ‘Does not apply’ options, coded ‘−88’ and ‘−99’ respectively, and an example is Q26A-G (WellBQ, 2021). These items would skew the scores, and so such responses were discarded. However, they may be important in other studies. The WellBQ also lacks a recommended threshold for what is considered ‘good’ or ‘healthy’ for each factor, category, or the tool overall. These issues could be addressed in future research. Lastly, the WellBQ has not been applied to a broad workforce. Three existing studies focus on Head Start staff and clinicians. As such, no established benchmark exists to anchor WellBQ scoring (Farewell et al., 2024; Lee et al., 2025; Powers et al., 2024).

### 4.3. Limitations

One limitation of this study was limited demographic variables, including only age, income, education, and gender. Future work should analyze additional WFH-related variables (e.g., work type, occupational level, tenure, household type, work and family schedules). Additionally, demographics were grouped to simplify analyses, but groupings were unbalanced due to the recruited sample (only 36 individuals without a college degree). Lastly, we lack insight into the response rate, given that our tool was available online for 6 wk in a database accessible to >83,000 individuals who we did not actively recruit.

## 5. Conclusion

Moving forward, work modalities will continue evolving. Based on our study, two major suggestions arose. First, different demographics have different well-being needs. Individuals with higher income and education scored well on job-related well-being metrics but suffer in terms of overall health. Second, there is a need for further WellBQ testing to determine if the scoring inconsistencies of high vs low being anchored differently across questions are truly relevant. In addition, targeted usage of individual sections (or question sets) could be applicable and was not explored. There is a timely need for employer efforts to offer job-related support to those of lower income or lower education. Employers should be strategic about implementing relevant resources for those who may be disadvantaged, as well as resources for those who might be sacrificing overall health for productivity.

## Supplementary Material

Supplemental appendices containing tables

Supplemental data for this article can be accessed online at https://doi.org/10.1080/24725838.2025.2524778.

## OCCUPATIONAL APPLICATIONS

The COVID-19 pandemic has significantly impacted workers and work. As Work from Home (WFH) modalities become more widely utilized, employers and workers alike face new benefits and challenges. The key aim of this report is to better understand the impact of WFH on the well-being of workers and identify potential risks of the WFH environment for this population subset. Using the NIOSH Worker Well-Being Questionnaire (WellBQ) tool, we quantified worker’s well-being across five categories defined by the tool. Scores from individual items within these categories were then evaluated across different demographic factors (age, gender, education level, income, and WFH level) to better understand the implications of the WFH modality for the diverse WFH workforce. Evaluating well-being against the demographic scores may shed light on groups that need additional support, resources and tools to perform their work and remain in good health.
