# Supplementary material for: Surveying Holistic Well-Being for Work from Home Employees: Insights for Organizational Practices
Source: IISE Trans Occup Ergon Hum Factors. Author manuscript; Available in PMC 2026 Jun 25. (PMC13296678; doi:10.1080/24725838.2025.2524778)
Supplement: Supplemental appendices containing tables [file NIHMS2148483-supplement-Supplemental_appendices_containing_tables.pdf]

Appendix A: Demographic Distribution

Table 1: Participant count in different demographic variables

**Age**	**18-24**	**25-34**	**35-44**	**45-54**	**55-64**	**65-74**	**75-84**	**85+**
**Count**	24	74	73	41	30	3	1	0
**Education Level**	**Less than High School**	**High School**	**Some College**	**Bachelor's or higher**				
**Count**	0	4	32	209				
**Sex**	**Male**	**Female**						
**Count**	54	190						
**Income**	**<$20,000USD**	**$20,000-$34,999USD**	**$35,000-$49,000USD**	**$50,000 - $74,999USD**	**$75,000 - $99,999USD**	**$100,000 to $149,999USD**	**$150,000 to $199,999USD**	**$200,000 USD or more**
**Count**	5	19	26	50	34	55	32	22
**WFH Days**	**1**	**2**	**3**	**4**	**5**			
**Count**	14	31	46	43	112			

Appendix B: Summary Statistic Tables of Sections 1-5

Table 1: Summary statistics of Section 1: Work evaluation & experience with correlations. Statistically significant results are highlighted in yellow

Variable (min, max)	Mean (SD)	Age			Income			Education			WFH Level			Gender		
		Low (171)	High (75)	Point-Biserial Correlation	Low (143)	High (100)	Point-Biserial Correlation	Low (36)	High (209)	Point-Biserial Correlation	Low (91)	High (155)	Point-Biserial Correlation	Male (54)	Female (190)	Point-Biserial Correlation
Job Satisfaction (1-4)	3.35 (0.6)	3.31 (0.59)	3.45 (0.62)	*r*(244)=0.1, *p*=0.1	3.2 (0.6)	3.47 (0.57)	*r*(241)=0.23, *p*=0.0004	3.3 (0.62)	3.36 (0.6)	*r*(243)=0.03, *p*=0.59	3.29 (0.58)	3.39 (0.61)	*r*(244)=0.08, *p*=0.23	3.35 (0.58)	3.35 (0.61)	*r*(242)=0, *p*=0.95
Wage Satisfaction (1-4)	2.91 (0.79)	2.82 (0.8)	3.12 (0.73)	*r*(243)=0.17, *p*=0.008	2.65 (0.81)	3.1 (0.73)	*r*(240)=0.28, *p*=0	2.94 (0.86)	2.91 (0.78)	*r*(242)=-0.01, *p*=0.83	2.9 (0.74)	2.92 (0.82)	*r*(243)=0.02, *p*=0.79	3.01 (0.68)	2.88 (0.82)	*r*(241)=-0.07, *p*=0.29
Benefits Satisfaction (1-4)	3.22 (1.04)	3.17 (1.02)	3.34 (1.08)	*r*(244)=0.08, *p*=0.24	3.02 (1.1)	3.37 (0.96)	*r*(241)=0.17, *p*=0.008	2.8 (1.28)	3.29 (0.98)	*r*(243)=0.17, *p*=0.01	3.23 (0.95)	3.22 (1.09)	*r*(244)=0, *p*=0.97	3.25 (0.82)	3.21 (1.1)	*r*(242)=-0.02, *p*=0.79
Advancement Satisfaction (1-4)	2.84 (0.85)	2.81 (0.83)	2.9 (0.9)	*r*(244)=0.05, *p*=0.43	2.75 (0.88)	2.91 (0.81)	*r*(241)=0.1, *p*=0.13	3 (0.95)	2.8 (0.83)	*r*(243)=-0.08, *p*=0.21	2.81 (0.8)	2.85 (0.88)	*r*(244)=0.03, *p*=0.69	2.92 (0.82)	2.81 (0.86)	*r*(242)=-0.06, *p*=0.38
Supervisor Support (1-4)	3.37 (0.94)	3.39 (0.82)	3.33 (1.16)	*r*(244)=-0.03, *p*=0.66	3.26 (1.01)	3.47 (0.87)	*r*(241)=0.11, *p*=0.078	3.11 (1.23)	3.42 (0.87)	*r*(243)=0.12, *p*=0.07	3.4 (0.82)	3.35 (1)	*r*(244)=-0.03, *p*=0.68	3.35 (1.08)	3.38 (0.9)	*r*(242)=0.01, *p*=0.82
Coworker Support (1-4)	3.32 (0.95)	3.29 (0.94)	3.41 (0.97)	*r*(244)=0.06, *p*=0.36	3.08 (1.12)	3.5 (0.76)	*r*(241)=0.22, *p*=0.0006	3.16 (1.15)	3.35 (0.91)	*r*(243)=0.07, *p*=0.28	3.3 (1.01)	3.34 (0.91)	*r*(244)=0.02, *p*=0.79	3.22 (0.88)	3.35 (0.97)	*r*(242)=0.06, *p*=0.36
Job Security (1-4)	3.17 (0.83)	3.13 (0.83)	3.26 (0.84)	*r*(244)=0.07, *p*=0.25	3.09 (0.86)	3.23 (0.81)	*r*(241)=0.08, *p*=0.2	2.97 (0.99)	3.21 (0.8)	*r*(243)=0.1, *p*=0.12	3.21 (0.87)	3.14 (0.81)	*r*(244)=-0.04, *p*=0.52	3.11 (0.9)	3.19 (0.82)	*r*(242)=0.04, *p*=0.52
Autonomy (1-4)	3.32 (0.8)	3.2 (0.83)	3.58 (0.65)	*r*(244)=0.22, *p*=0.001	3.14 (0.8)	3.45 (0.78)	*r*(241)=0.19, *p*=0.003	3.19 (0.98)	3.34 (0.76)	*r*(243)=0.07, *p*=0.3	3.16 (0.82)	3.41 (0.77)	*r*(244)=0.15, *p*=0.02	3.12 (0.97)	3.37 (0.74)	*r*(242)=0.13, *p*=0.04
Work Overload (1-4)	2.44 (0.94)	2.45 (0.97)	2.41 (0.88)	*r*(244)=-0.02, *p*=0.75	2.47 (0.98)	2.43 (0.92)	*r*(241)=-0.02, *p*=0.77	2.58 (1.2)	2.41 (0.9)	*r*(243)=-0.06, *p*=0.33	2.42 (0.92)	2.45 (0.96)	*r*(244)=0.01, *p*=0.85	2.29 (1.02)	2.48 (0.92)	*r*(242)=0.08, *p*=0.19
Work Fatigue (1-7)	3.51 (1.31)	3.41 (1.29)	3.73 (1.34)	*r*(244)=0.11, *p*=0.081	3.4 (1.34)	3.6 (1.29)	*r*(241)=0.08, *p*=0.23	3.44 (1.48)	3.52 (1.29)	*r*(243)=0.02, *p*=0.75	3.7 (1.37)	3.4 (1.27)	*r*(244)=-0.11, *p*=0.08	3.55 (1.25)	3.48 (1.33)	*r*(242)=-0.02, *p*=0.73
Positive affect (1-7)	4.95 (1.12)	4.91 (1.14)	5.06 (1.06)	*r*(242)=0.06, *p*=0.34	4.73 (1.14)	5.13 (1.05)	*r*(239)=0.18, *p*=0.006	4.63 (1.35)	5.01 (1.07)	*r*(241)=0.12, *p*=0.06	5.02 (1.1)	4.91 (1.13)	*r*(242)=-0.05, *p*=0.48	4.72 (1.3)	5.02 (1.06)	*r*(240)=0.11, *p*=0.08
Negative affect (1-7)	4.62 (1.21)	4.5 (1.21)	4.87 (1.18)	*r*(243)=0.14, *p*=0.029	4.42 (1.2)	4.77 (1.19)	*r*(240)=0.14, *p*=0.027	4.68 (1.36)	4.6 (1.19)	*r*(242)=-0.02, *p*=0.74	4.5 (1.24)	4.68 (1.19)	*r*(243)=0.07, *p*=0.27	4.47 (1.27)	4.65 (1.2)	*r*(241)=0.06, *p*=0.33
Work Meaning (1-4)	3.33 (0.64)	3.24 (0.66)	3.52 (0.54)	*r*(244)=0.2, *p*=0.002	3.23 (0.67)	3.41 (0.61)	*r*(241)=0.14, *p*=0.035	3.23 (0.76)	3.35 (0.62)	*r*(243)=0.06, *p*=0.31	3.34 (0.66)	3.32 (0.63)	*r*(244)=-0.02, *p*=0.81	3.17 (0.74)	3.38 (0.6)	*r*(242)=0.13, *p*=0.04
Engagement (1-7)	4.86 (1.11)	4.77 (1.14)	5.07 (1.01)	*r*(243)=0.12, *p*=0.055	4.8 (1.08)	4.92 (1.13)	*r*(241)=0.05, *p*=0.4	4.78 (1.44)	4.88 (1.04)	*r*(242)=0.03, *p*=0.63	4.99 (1.03)	4.79 (1.15)	*r*(243)=-0.09, *p*=0.17	4.73 (1.28)	4.91 (1.05)	*r*(241)=0.07, *p*=0.29

Table 2: Summary statistics of Section 2: Policies and culture with correlations. Statistically significant results are highlighted in yellow

**Variable (min, max)**	**Mean (SD) n=246**	**Age**			**Income**			**Education**			**WFH Level**			**Gender**		
		Low (171)	High (75)	Point-Biserial Correlation	Low (100)	High (143)	Point-Biserial Correlation	Low (36)	High (209)	Point-Biserial Correlation	Low (91)	High (155)	Point-Biserial Correlation	Male (54)	Female (190)	Point-Biserial Correlation
Management Trust (1-4)	2.96 (0.95)	2.94 (0.92)	3.01 (1.04)	$r(244)=0.03, p=0.62$	2.98 (1.07)	2.96 (0.88)	$r(241)=-0.02, p=0.9$	2.91 (1.18)	2.97 (0.92)	$r(243)=0.02, p=0.73$	3.05 (0.88)	2.91 (0.99)	$r(244)=-0.06, p=0.27$	3.01 (1.01)	2.95 (0.94)	$r(242)=-0.02, p=0.68$
Work to Non-work Conflict (1-7)	4.11 (1.33)	4.14 (1.3)	4.06 (1.42)	$r(244)=-0.02, p=0.69$	4.17 (1.49)	4.08 (1.19)	$r(241)=0.01, p=0.61$	4.05 (1.75)	4.11 (1.25)	$r(243)=0.01, p=0.79$	4.02 (1.28)	4.17 (1.37)	$r(244)=0.05, p=0.39$	4.05 (1.45)	4.11 (1.3)	$r(242)=0.01, p=0.77$
Non-work to work Conflict (1-7)	4.4 (1.38)	4.33 (1.39)	4.56 (1.35)	$r(244)=0.07, p=0.23$	4.53 (1.45)	4.32 (1.32)	$r(241)=0, p=0.26$	4.88 (1.63)	4.31 (1.32)	$r(243)=-0.14, p=0.02$	4.71 (1.11)	4.21 (1.49)	$r(244)=-0.17, p=0$	4.37 (1.54)	4.4 (1.34)	$r(242)=0, p=0.89$
Supportive Work Culture (1-4)	3.23 (0.67)	3.23 (0.6)	3.22 (0.81)	$r(242)=0, p=0.88$	3.16 (0.69)	3.29 (0.64)	$r(239)=0, p=0.13$	3.13 (0.74)	3.24 (0.66)	$r(241)=0.06, p=0.34$	3.22 (0.62)	3.23 (0.7)	$r(242)=0, p=0.9$	3.24 (0.64)	3.23 (0.68)	$r(240)=0, p=0.92$
Health Culture at Work (1-4)	3.07 (0.91)	3.09 (0.85)	3.02 (1.04)	$r(244)=-0.03, p=0.59$	2.92 (0.96)	3.18 (0.85)	$r(241)=-0.04, p=0.02$	2.75 (1)	3.12 (0.89)	$r(243)=0.14, p=0.02$	3.1 (0.75)	3.05 (0.99)	$r(244)=-0.02, p=0.68$	3.15 (0.81)	3.05 (0.94)	$r(242)=-0.04, p=0.47$
Job Benefits (0-14)	8.58 (3.74)	8.41 (3.75)	8.98 (3.7)	$r(239)=0.07, p=0.27$	7.52 (3.9)	9.4 (3.39)	$r(236)=0.11, p=0$	7.09 (4.36)	8.82 (3.59)	$r(238)=0.15, p=0.01$	8.47 (3.7)	8.65 (3.77)	$r(239)=0.02, p=0.72$	7.79 (3.77)	8.81 (3.73)	$r(237)=0.11, p=0.08$
Health Program at work (0-7)	3.1 (2.24)	3.08 (2.11)	3.17 (2.53)	$r(244)=0.01, p=0.76$	2.92 (2.13)	3.28 (2.3)	$r(241)=0.05, p=0.21$	2.72 (2.03)	3.17 (2.28)	$r(243)=0.07, p=0.26$	3.19 (2.36)	3.05 (2.18)	$r(244)=-0.03, p=0.63$	2.88 (2.19)	3.17 (2.27)	$r(242)=0.05, p=0.41$
Workplace/Schedule Flexibility (1-4)	3.01 (0.86)	3.02 (0.9)	3 (0.75)	$r(244)=0, p=0.9$	2.93 (0.91)	3.07 (0.82)	$r(241)=-0.01, p=0.2$	2.72 (0.88)	3.06 (0.85)	$r(243)=0.14, p=0.02$	3.01 (0.78)	3.01 (0.9)	$r(244)=0, p=0.94$	3.04 (0.73)	3.01 (0.89)	$r(242)=-0.01, p=0.78$

Table 3: Summary statistics of Section 3: Workplace physical safety climate with correlations. Statistically significant results are highlighted in yellow

**Variable (min, max)**	**Mean (SD) n=246**	**Age**			**Income**			**Education**			**WFH Level**			**Gender**		
		Low (171)	High (75)	Point-Biserial Correlation	Low (100)	High (143)	Point-Biserial Correlation	Low (36)	High (209)	Point-Biserial Correlation	Low (91)	High (155)	Point-Biserial Correlation	Male (54)	Female (190)	Point-Biserial Correlation
Overall Workplace Safety (1-4)	3.63 (0.63)	3.57 (0.65)	3.74 (0.57)	$r(244)=0.12, p=0.06$	3.54 (0.73)	3.69 (0.55)	$r(241)=0.12, p=0.06$	3.5 (0.77)	3.65 (0.6)	$r(243)=0.08, p=0.18$	3.52 (0.63)	3.69 (0.63)	$r(244)=0.12, p=0.05$	3.57 (0.68)	3.65 (0.62)	$r(242)=0.05, p=0.42$
Sexual Harassment (0-1)	0.96 (0.18)	0.95 (0.21)	0.98 (0.11)	$r(242)=0.08, p=0.21$	0.93 (0.25)	0.98 (0.11)	$r(239)=0.14, p=0.02$	0.82 (0.38)	0.98 (0.11)	$r(241)=0.29, p=0$	0.95 (0.2)	0.96 (0.17)	$r(242)=0.02, p=0.65$	0.92 (0.26)	0.97 (0.16)	$r(240)=0.1, p=0.1$
Physical Violence (0-1)	0.95 (0.19)	0.94 (0.23)	1 (0)	$r(243)=0.13, p=0.03$	0.92 (0.27)	0.98 (0.11)	$r(240)=0.16, p=0.01$	0.88 (0.32)	0.97 (0.16)	$r(242)=0.15, p=0.02$	0.93 (0.24)	0.97 (0.15)	$r(243)=0.09, p=0.13$	0.9 (0.29)	0.97 (0.14)	$r(241)=0.15, p=0.01$
Workplace Safety Climate (1-4)	2.19 (1.36)	2.31 (1.31)	1.91 (1.42)	$r(241)=-0.13, p=0.04$	2.28 (1.34)	2.14 (1.36)	$r(238)=-0.05, p=0.42$	2.33 (1.38)	2.17 (1.35)	$r(240)=-0.04, p=0.51$	2.64 (1.16)	1.92 (1.4)	$r(241)=-0.25, p=0$	2.67 (1.21)	2.06 (1.37)	$r(239)=-0.18, p=0$
Physical Work Environment Satisfaction (1-4)	2.95 (0.64)	2.9 (0.61)	3.06 (0.69)	$r(241)=0.11, p=0.08$	2.91 (0.58)	2.97 (0.69)	$r(238)=0.04, p=0.51$	3.06 (0.6)	2.92 (0.65)	$r(240)=-0.07, p=0.26$	3.01 (0.6)	2.91 (0.66)	$r(241)=-0.07, p=0.25$	3.01 (0.63)	2.92 (0.65)	$r(239)=-0.05, p=0.37$
Discrimination (1-4)	3.58 (0.59)	3.52 (0.61)	3.71 (0.49)	$r(243)=0.14, p=0.03$	3.46 (0.65)	3.66 (0.52)	$r(240)=0.16, p=0.01$	3.31 (0.84)	3.63 (0.52)	$r(242)=0.19, p=0$	3.54 (0.63)	3.6 (0.56)	$r(243)=0.04, p=0.44$	3.54 (0.7)	3.59 (0.55)	$r(241)=0.03, p=0.6$
Work Related Bullying (0-1)	0.82 (0.3)	0.82 (0.3)	0.83 (0.31)	$r(242)=0.02, p=0.68$	0.79 (0.33)	0.84 (0.28)	$r(239)=0.07, p=0.22$	0.7 (0.34)	0.84 (0.29)	$r(241)=0.16, p=0.01$	0.78 (0.33)	0.85 (0.28)	$r(242)=0.11, p=0.07$	0.82 (0.29)	0.82 (0.3)	$r(240)=0, p=0.99$

Table 4: Summary statistics of Section 4: Health status with correlations. Statistically significant results are highlighted in yellow

Variable (min, max)	Mean (SD) n=246	Age			Income			Education			WFH Level			Gender		
		Low (171)	High (75)	Point-Biserial Correlation	Low (100)	High (143)	Point-Biserial Correlation	Low (36)	High (209)	Point-Biserial Correlation	Low (91)	High (155)	Point-Biserial Correlation	Male (54)	Female (190)	Point-Biserial Correlation
Overall Health (1-5)	2.6 (0.9)	2.64 (0.87)	2.52 (0.94)	*r*(244)=-0.06, *p*=0.32	2.75 (0.93)	2.48 (0.85)	*r*(241)=-0.14, *p*=0.03	2.94 (0.92)	2.55 (0.88)	*r*(243)=-0.15, *p*=0.02	2.56 (0.93)	2.63 (0.88)	*r*(244)=0.03, *p*=0.55	2.57 (0.9)	2.61 (0.9)	*r*(242)=0.01, *p*=0.77
Days of poor physical Health (0-30)	3.93 (6.13)	3.91 (5.87)	3.95 (6.72)	*r*(233)=0, *p*=0.96	4.15 (6.12)	3.68 (6.06)	*r*(230)=-0.03, *p*=0.57	4.78 (6.07)	3.81 (6.16)	*r*(232)=-0.05, *p*=0.4	2.55 (3.42)	4.72 (7.14)	*r*(233)=0.17, *p*=0.01	3.09 (5.01)	4.2 (6.43)	*r*(231)=0.07, *p*=0.25
Days of poor mental health (0-30)	6.42 (7.75)	7.44 (8.26)	4.2 (5.96)	*r*(231)=-0.19, *p*=0	8.41 (9.07)	4.96 (6.2)	*r*(228)=-0.22, *p*=0	8.61 (8.93)	6.12 (7.55)	*r*(230)=-0.1, *p*=0.1	6.51 (7.18)	6.37 (8.08)	*r*(231)=0, *p*=0.9	7.08 (9.3)	6.29 (7.36)	*r*(229)=-0.04, *p*=0.54
Healthy Diet (1-6)	3.72 (1.13)	3.8 (1.1)	3.53 (1.17)	*r*(220)=-0.1, *p*=0.11	3.89 (0.97)	3.57 (1.23)	*r*(217)=-0.14, *p*=0.04	3.87 (1.36)	3.68 (1.08)	*r*(219)=-0.06, *p*=0.37	3.79 (0.97)	3.67 (1.22)	*r*(220)=-0.05, *p*=0.45	3.79 (1.06)	3.69 (1.15)	*r*(218)=-0.03, *p*=0.57
Sleep Hours (0-1)	0.28 (0.45)	0.29 (0.45)	0.28 (0.45)	*r*(244)=-0.01, *p*=0.84	0.33 (0.47)	0.25 (0.43)	*r*(241)=-0.07, *p*=0.23	0.47 (0.5)	0.25 (0.43)	*r*(243)=-0.16, *p*=0.01	0.3 (0.46)	0.27 (0.44)	*r*(244)=-0.03, *p*=0.61	0.35 (0.48)	0.27 (0.44)	*r*(242)=-0.07, *p*=0.27
Sleepy at work (1-5)	2.91 (0.95)	2.98 (0.97)	2.76 (0.89)	*r*(244)=-0.11, *p*=0.08	3.08 (0.96)	2.79 (0.93)	*r*(241)=-0.14, *p*=0.02	3.02 (0.97)	2.9 (0.95)	*r*(243)=-0.04, *p*=0.47	2.84 (0.91)	2.96 (0.97)	*r*(244)=0.05, *p*=0.36	2.77 (1.02)	2.96 (0.93)	*r*(242)=0.08, *p*=0.21
Cognitive Functioning Limitations (1-4)	1.8 (1)	1.88 (1.06)	1.62 (0.85)	*r*(244)=-0.11, *p*=0.07	2.13 (1.09)	1.58 (0.88)	*r*(241)=-0.26, *p*=0	2.22 (1.19)	1.73 (0.95)	*r*(243)=-0.17, *p*=0.01	1.87 (1.03)	1.76 (0.99)	*r*(244)=-0.05, *p*=0.38	1.83 (1.09)	1.8 (0.98)	*r*(242)=-0.01, *p*=0.83
Work Limitation (1-4)	1.47 (0.81)	1.46 (0.83)	1.5 (0.77)	*r*(244)=0.02, *p*=0.73	1.67 (0.89)	1.34 (0.73)	*r*(241)=-0.19, *p*=0	1.8 (0.98)	1.42 (0.77)	*r*(243)=-0.16, *p*=0.01	1.56 (0.81)	1.43 (0.81)	*r*(244)=-0.07, *p*=0.24	1.5 (0.79)	1.47 (0.82)	*r*(242)=-0.01, *p*=0.87
Work-Related Injury (0-1)	0.04 (0.19)	0.04 (0.19)	0.04 (0.19)	*r*(242)=0, *p*=0.98	0.05 (0.22)	0.03 (0.18)	*r*(239)=-0.03, *p*=0.56	0.11 (0.31)	0.02 (0.16)	*r*(241)=-0.14, *p*=0.02	0.05 (0.23)	0.03 (0.17)	*r*(242)=-0.05, *p*=0.38	0.09 (0.29)	0.02 (0.14)	*r*(240)=-0.15, *p*=0.01
Injury Consequence (0-1)	0.02 (0.16)	0.02 (0.15)	0.04 (0.19)	*r*(243)=0.04, *p*=0.48	0.03 (0.17)	0.02 (0.16)	*r*(240)=0, *p*=0.92	0.02 (0.16)	0.02 (0.16)	*r*(242)=0, *p*=0.97	0.04 (0.2)	0.01 (0.13)	*r*(243)=-0.07, *p*=0.27	0.03 (0.19)	0.02 (0.16)	*r*(241)=-0.02, *p*=0.68
Insomnia (0-1)	0.34 (0.47)	0.32 (0.46)	0.4 (0.49)	*r*(243)=0.07, *p*=0.25	0.37 (0.48)	0.33 (0.47)	*r*(240)=-0.04, *p*=0.53	0.54 (0.5)	0.31 (0.46)	*r*(242)=-0.16, *p*=0.01	0.26 (0.44)	0.39 (0.49)	*r*(243)=0.13, *p*=0.04	0.37 (0.48)	0.34 (0.47)	*r*(241)=-0.02, *p*=0.72
Alcohol Consumption (0-1)	0.01 (0.12)	0.01 (0.13)	0.01 (0.11)	*r*(239)=-0.01, *p*=0.79	0.02 (0.14)	0.01 (0.11)	*r*(237)=-0.02, *p*=0.71	0.02 (0.17)	0.01 (0.11)	*r*(239)=-0.04, *p*=0.53	0.01 (0.1)	0.01 (0.13)	*r*(239)=0.03, *p*=0.62	0.07 (0.26)	0 (0)	*r*(239)=-0.24, *p*=0
Risky Drinking (0-1)	0.48 (0.5)	0.52 (0.5)	0.38 (0.49)	*r*(243)=-0.12, *p*=0.05	0.53 (0.5)	0.44 (0.49)	*r*(240)=-0.09, *p*=0.15	0.5 (0.5)	0.47 (0.5)	*r*(242)=-0.01, *p*=0.79	0.53 (0.5)	0.45 (0.49)	*r*(243)=-0.07, *p*=0.22	0.54 (0.5)	0.46 (0.49)	*r*(241)=-0.06, *p*=0.28
Chronic Health Conditions (0-9)	2.02 (1.67)	1.79 (1.66)	2.6 (1.57)	*r*(237)=0.21, *p*=0	2.14 (1.91)	1.92 (1.46)	*r*(234)=-0.06, *p*=0.33	3.52 (2.16)	1.78 (1.44)	*r*(236)=-0.36, *p*=0	1.89 (1.7)	2.1 (1.66)	*r*(237)=0.06, *p*=0.36	2.01 (1.99)	2.04 (1.57)	*r*(235)=0, *p*=0.91
Overall Stress (1-7)	3.97 (1.12)	4.13 (1.16)	3.62 (0.93)	*r*(239)=-0.21, *p*=0	4.29 (1.21)	3.76 (0.98)	*r*(236)=-0.23, *p*=0	4.19 (1.44)	3.95 (1.04)	*r*(238)=-0.07, *p*=0.23	3.9 (1.16)	4.01 (1.09)	*r*(239)=0.05, *p*=0.43	3.94 (1.23)	4 (1.08)	*r*(237)=0.01, *p*=0.77
Poor Mental Health (1-4)	1.7 (0.66)	1.78 (0.66)	1.52 (0.62)	*r*(240)=-0.18, *p*=0	1.94 (0.73)	1.54 (0.55)	*r*(237)=-0.29, *p*=0	2.06 (0.78)	1.64 (0.62)	*r*(239)=-0.22, *p*=0	1.75 (0.66)	1.67 (0.65)	*r*(240)=-0.05, *p*=0.38	1.77 (0.77)	1.68 (0.62)	*r*(238)=-0.05, *p*=0.37
Physical Activity (0-7)	3.97 (1.77)	4.05 (1.82)	3.79 (1.68)	*r*(239)=-0.06, *p*=0.29	3.85 (1.85)	4.01 (1.72)	*r*(236)=0.04, *p*=0.49	3.95 (1.92)	3.96 (1.75)	*r*(238)=0, *p*=0.97	3.86 (1.83)	4.03 (1.75)	*r*(239)=0.04, *p*=0.46	3.66 (1.6)	4.04 (1.82)	*r*(237)=0.08, *p*=0.17
Tobacco Use (0-5)	0.18 (0.52)	0.23 (0.6)	0.06 (0.25)	*r*(242)=-0.14, *p*=0.03	0.33 (0.66)	0.07 (0.37)	*r*(239)=-0.23, *p*=0	0.52 (0.84)	0.12 (0.42)	*r*(241)=-0.27, *p*=0	0.25 (0.6)	0.13 (0.47)	*r*(242)=-0.1, *p*=0.1	0.44 (0.86)	0.1 (0.35)	*r*(240)=-0.26, *p*=0
Productivity (1-7)	2.87 (1.28)	3.03 (1.37)	2.51 (0.95)	*r*(239)=-0.18, *p*=0	3.19 (1.31)	2.63 (1.2)	*r*(236)=-0.21, *p*=0	3.14 (1.41)	2.82 (1.26)	*r*(238)=-0.08, *p*=0.17	2.94 (1.31)	2.83 (1.27)	*r*(239)=-0.04, *p*=0.52	3.11 (1.44)	2.8 (1.23)	*r*(237)=-0.09, *p*=0.13

Table 5: Summary statistics of Section 5: Home Community & Society with correlations. Statistically significant results are highlighted in yellow

Variable (min, max)	Mean (SD) n=246	Age			Income			Education			WFH Level			Gender		
		Low (171)	High (75)	Point-Biserial Correlation	Low (100)	High (143)	Point-Biserial Correlation	Low (36)	High (209)	Point-Biserial Correlation	Low (91)	High (155)	Point-Biserial Correlation	Male (54)	Female (190)	Point-Biserial Correlation
Life Satisfaction (1-4)	3.16 (0.71)	3.14 (0.69)	3.21 (0.75)	*r*(243)=0.04, *p*=0.47	2.94 (0.76)	3.32 (0.62)	*r*(240)=0.26, *p*=0	2.94 (0.86)	3.2 (0.68)	*r*(242)=0.12, *p*=0.05	3.15 (0.71)	3.16 (0.72)	*r*(243)=0.01, *p*=0.87	3.07 (0.74)	3.19 (0.71)	*r*(241)=0.06, *p*=0.3
Support outside of work (1-4)	3.18 (0.78)	3.19 (0.77)	3.14 (0.81)	*r*(244)=-0.03, *p*=0.63	2.98 (0.81)	3.32 (0.72)	*r*(241)=0.21, *p*=0	2.8 (0.85)	3.24 (0.75)	*r*(243)=0.19, *p*=0	3.15 (0.8)	3.2 (0.77)	*r*(244)=0.02, *p*=0.66	2.94 (0.87)	3.24 (0.74)	*r*(242)=0.16, *p*=0.01
Financial Insecurity (1-4)	2.71 (0.87)	2.65 (0.88)	2.86 (0.83)	*r*(243)=0.11, *p*=0.08	2.41 (0.86)	2.93 (0.82)	*r*(240)=0.28, *p*=0	2.48 (1.05)	2.75 (0.83)	*r*(242)=0.1, *p*=0.09	2.68 (0.85)	2.73 (0.88)	*r*(243)=0.02, *p*=0.69	2.68 (0.88)	2.72 (0.87)	*r*(241)=0.02, *p*=0.75
Activities outside of work (0-7)	2.32 (1.9)	2.61 (1.97)	1.63 (1.53)	*r*(243)=-0.23, *p*=0	2.83 (2.13)	1.97 (1.66)	*r*(240)=-0.21, *p*=0	3 (2.17)	2.21 (1.83)	*r*(242)=-0.14, *p*=0.02	2.43 (2.16)	2.25 (1.74)	*r*(243)=-0.04, *p*=0.46	2.44 (2.3)	2.29 (1.79)	*r*(241)=-0.03, *p*=0.6
